# Hand-suture versus stapling for closure of loop ileostomy: HASTA-Trial: a study rationale and design for a randomized controlled trial

**DOI:** 10.1186/1745-6215-12-34

**Published:** 2011-02-08

**Authors:** Thorsten Löffler, Christoph M Seiler, Inga Rossion, Thomas Kijak, Oliver Thomusch, Renè Hodina, Matthias Krüger, Thomas Simon, Thomas Bruckner, Meinhard Kieser, Markus W Büchler, Jürgen Weitz

**Affiliations:** 1 Department of General, Visceral and Transplantation Surgery, University of Heidelberg, Heidelberg, Germany; 2 Study Center of the German Surgical Society, University of Heidelberg, Heidelberg, Germany; 3 Institute of Medical Biometry and Informatics, University of Heidelberg, Heidelberg, Germany; 4 Department of General and Visceral Surgery, Robert Bosch Hospital, Stuttgart, Germany; 5 Department of General and Visceral Surgery, University of Freiburg, Freiburg, Germany; 6 Department of General, Visceral and Thoracic Surgery, Klinik am Steinenberg, Reut lingen, Germany; 7 Magdeburg Hospital gGmbH, Magdeburg, Germany; 8 Department of General and Visceral Surgery, Krankenhaus Sinsheim, Germany

## Abstract

**Background:**

Colorectal cancer is the second most common tumor in developed countries, with a lifetime prevalence of 5%. About one third of these tumors are located in the rectum. Surgery in terms of low anterior resection with mesorectal excision is the central element in the treatment of rectal cancer being the only option for definite cure. Creating a protective diverting stoma prevents complications like anastomotic failure and meanwhile is the standard procedure. Bowel obstruction is one of the main and the clinically and economically most relevant complication following closure of loop ileostomy. The best surgical technique for closure of loop ileostomy has not been defined yet.

**Methods/Design:**

A study protocol was developed on the basis of the only randomized controlled mono-center trial to solve clinical equipoise concerning the optimal surgical technique for closure of loop ileostomy after low anterior resection due to rectal cancer.

The HASTA trial is a multi-center pragmatic randomized controlled surgical trial with two parallel groups to compare hand-suture versus stapling for closure of loop ileostomy. It will include 334 randomized patients undergoing closure of loop ileostomy after low anterior resection with protective ileostomy due to rectal cancer in approximately 20 centers consisting of German hospitals of all level of health care. The primary endpoint is the rate of bowel obstruction within 30 days after ileostomy closure. In addition, a set of surgical and general variables including quality of life will be analyzed with a follow-up of 12 months. An investigators meeting with a practical session will help to minimize performance bias and enforce protocol adherence. Centers are monitored centrally as well as on-site before and during recruitment phase to assure inclusion, treatment and follow up according to the protocol.

**Discussion:**

Aim of the HASTA trial is to evaluate the efficacy of hand-suture versus stapling for closure of loop ileostomy in patients with rectal cancer.

**Trial registration:**

German Clinical Trial Register Number: DRKS00000040

## Background

### Background and rationale

Colorectal cancer is the second most common tumor in developed countries, with a lifetime prevalence of 5% [1,2]. Approximately 30% of these tumors are located in the rectum. Surgery forms the cornerstone in the treatment of rectal cancer, with the low anterior resection (LAR) with totally mesorectal excision being the standard procedure [3]. Today, a diverting protective stoma should be used until definite healing of the anastomosis is achieved [4,5]. So far, it remains still uncertain whether a loop ileostomy or a colostomy is preferable [6-8]. After a period of three months the ileo- or colostomy is subsequently closed and intestinal continuity is re-established. Due to the high prevalence of rectal cancer, this is a frequently performed procedure in surgical practice.

### Preliminary data

One randomized controlled trial (RCT) including 141 patients over 6 years has compared hand-sutured and stapled anastomosis for closure of loop ileostomy so far [9]. The results of this trial have shown a significantly higher rate of postoperative bowel obstruction (14% versus 3%, p = 0.0168) for patients who received hand-sutured anastomosis. However, there are severe methodological issues which impair the interpretation of this trial. First, the trial included a heterogeneous group of patients with many different underlying benign and malignant diseases. Second, it was performed in a single-center setting with only a low number of surgeons performing the investigated techniques. Therefore, the results reflect the situation of loop ileostomy closure at this institution rather than practice in general. Third, results of this trial are probably biased by learning curve effects of participating surgeons [10]. The authors report that two-thirds of bowel obstructions observed in the trial occurred in the first half of the study. In addition, all patients requiring re-operations had initial closure of the loop ileostomy performed by senior registrars (as opposed to consultant surgeons). Due to the methodological and clinical limitations of this trial a multi-center pragmatic trial is needed to confirm the observed findings.

### Objectives and hypotheses

The objective of the HASTA-Trial is to investigate whether there is a difference in rate of bowel obstruction one month after hand-suture as compared to stapling loop ileostomy closure. If p_HA_/p_HA _denotes the rate of occurrence of bowel obstruction within one month after ileostomy closure in the hand-suture group (HA)/stapler group (STA), then the following two-sided test problem is assessed: H_0_: p_HA _= p_STA _vs. H_1_: p_HA _≠ p_STA_.

## Methods/Design

### Study population and Trial group

Patients with history of low anterior resection (LAR) and creating of a protective loop ileostomy for rectal cancer who are planned for elective closure of loop ileostomy will be recruited for this trial.

The HASTA-Trial is conducted by a national study group consisting of certified bowel centers and other German hospitals of all level of health care. Trial design and management are under the responsibility of the Study Center of the German Surgical Society, biostatistical planning, data management and analysis is performed by the Institute of Medical Biometry and Informatics, University of Heidelberg.

#### Subject inclusion criteria

• Patients equal or older than 18 years scheduled for elective ileostomy closure after LAR

• Informed consent

#### Subject exclusion criteria

• Pathologic findings in routine preoperative diagnostic tests (e.g. anastomotic leakage) which do not allow a safe ileostomy closure

• Participation in another intervention-trial with interference of intervention and outcome of this study

• Expected lack of compliance

Centers are asked to document potential patients in a screening log. Thus, reasons for exclusion are documented.

### Sample size

The prior assumptions for sample size calculation are based on the results of Hasegawa et al. (2000) ([9]) and assume prevalence rates with respect to the primary endpoint of 3% in the stapler group and 14% in the hand-suture group. To detect this difference with the two-sided chi-square test at a type I error rate of (two-sided) with power, a sample size of n = 133 evaluable patients (treatment and follow up according to the protocol) per group is necessary. It can be expected that taking into account the covariate "skill of surgeon" in the analysis will increase the power as compared to the chi-square test. The drop out rate within one month after index operation is expected to be about 20% overall. Therefore, another total of 68 patients have to be randomized to obtain the required number of evaluable patients. The total sample size to be randomized is therefore 334 patients.

### Randomization and blinding

The randomization numbers will be allocated to the two groups in balanced permuted blocks and stratified by center using the web-based software "Randomizer" provided by the Institute of Medical Informatics, Statistics and Documentation of the Medical University of Graz (http://www.randomizer.at). This software allows choosing different randomization methods as well as different sets of parameters for the chosen method.

To avoid any potential of predicting the group allocation of future patients, the block length is fixed in a separate document that is withheld from the study site. In addition, persons with the right to randomize with the software described above do not have the right to read or edit the randomization design chosen within the software. The software stores the result of randomization and patient characteristics as well as the name of the person who randomized and the randomization date in a separate file, and only authorized persons can download this file. Patients are randomized the day before or on the day the surgical intervention is performed.

Patients are not blinded and blinding of the surgeon is unfeasible. Typical symptoms for the primary outcome, bowel obstruction, like productive vomiting, gastric tube placement, severe constipation are detected during regular patient care and documented in the medical record. Assessment according to trial criteria is done by investigators.

### Interventions

#### Treatment description

The following description of treatment is a recommendation and should be followed by all participating surgeons. Technique for hand-suture can be modified according to local standards. In contrast the stapling of an anastomosis must be performed as stated below.

#### Standardized mobilization of loop ileostomy

The operation is initiated with an oval skin incision around the ileostomy and temporarily closed by continuous suture to prevent further stool contamination. The loop ileostomy is then thoroughly mobilized from the subcutaneous layer and from the abdominal wall until it is loose and can be moved out of the peritoneal cavity.

#### Stapler group

**Figure 1:**
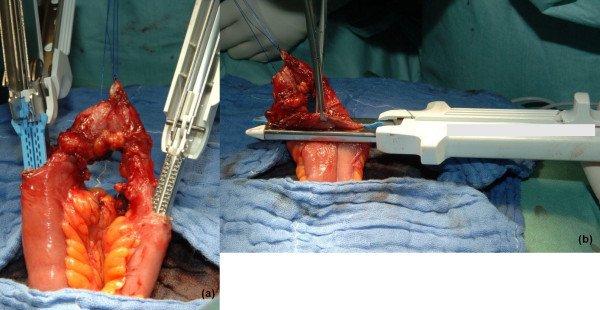
**Stapled side-to-side anastomosis before (a) and after (b) stapling**.

The TLC-75-stapler (Ethicon, Norderstedt) is brought into the two opened antimesenteric apexes of the intestinal shanks to facilitate side-to-side (functional end-to-end) anastomosis (Figure 1). The apex of the loop and the spout is cross-stapled with a refill of the TLC-stapler followed by overstitching the cross-stapled line with a Polydioxanon equivalent suture (USP 5-0/Ethicon, Norderstedt). The intestine is then put back into the peritoneal cavity. The abdominal wall is closed with interrupted sutures using Polyglactin equivalent sutures (USP 2). The subcutaneous tissue is not sutured and no subcutaneous drainage is used. The skin can be closed by either interrupted monofilament sutures or clips.

#### Hand-suture group

After thorough mobilisation the loop ileostomy is resected using two bowel clamps. An end-to-end anastomosis is performed as follows: a two-layer continuous suture using four Polydioxanon equivalent sutures (USP 5-0). The inner layer consists of a transmural suture, the outer layer of a sero-muscular suture. Alternatively, interrupted sutures may be performed depending on local standards. The abdominal wall and the skin are closed in the same way as for stapled closure.

#### Permitted and not permitted medication(s)/treatment(s)

No other method of ileostomy than the randomized and described technique in the protocol should be used for anastomosis. Any protocol violation has to be reported with a clear description.

The postoperative care is performed according to the principles and standard of the department.

### Outcomes (primary and secondary)

The primary endpoint is the occurrence of bowel obstruction within 30 days after ileostomy closure.

#### Assessment of the primary endpoint

Bowel obstruction is defined as productive vomiting or the need of gastric tube placement or absolute constipation with a duration of at least three days. This definition is based on the existing trial by Hasegawa [9] which provides the data for sample size calculation of the HASTA-Trial.

Secondary endpoints are the time needed to perform the procedure, wound infection, rate of re-operation due to anastomotic leakage of the ileostomy closure, time to first tolerance to solid food and first bowel movement, whichever of these occurred last, length of postoperative hospital stay, 30 days and 12 months mortality after ileostomy closure, rate of re-operation and re-hospitalization within one year due to bowel obstruction, costs of surgical procedure for the institution (including threads, stapler, time etc.), quality of life (EuroQol 5 D).

**Figure 2:**
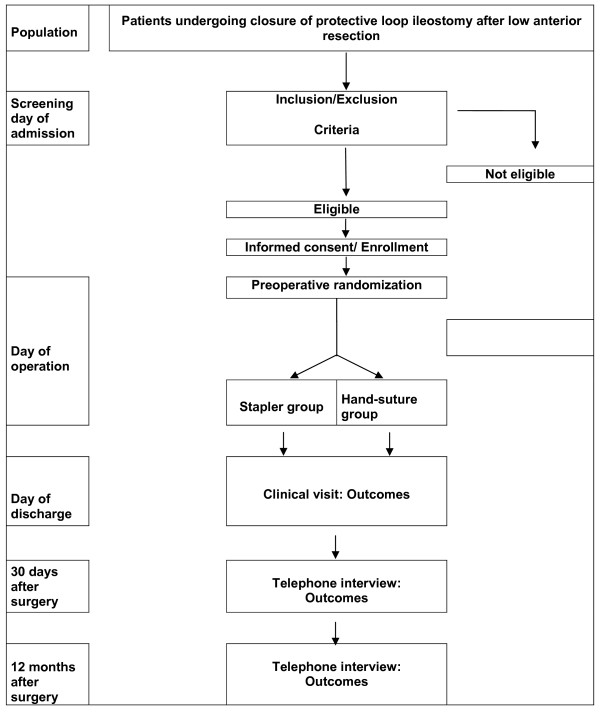
**Flowchart**.

Three out of, five visits are documented by the investigator in each center during in hospital phase (screening, intervention and discharge), followed by two telephone interviews for 30 days and 12 months follow up after operation (Table 1 and Figure 2).

**Table 1:** Course of examinations

Visit	1 (=Screening)	2 (Operation)	3 (day of discharge)	4 (30 days post OP)	5 (12 ± 1 months post OP)
Demographics and baseline clinical data	X				

Eligibility criteria	X				

Randomization, Surgical intervention		X			

Clinical visit/Follow-up			X	X	X

Quality of life (EuroQol 5 D)	X			X	X

Safety		X	X	X	X

### Data management and monitoring

#### Documentation

All protocol-required information collected during the trial must be entered by the investigator, or designated representative, in the case report form (CRF). A paper based CRF is used to collect the data. The investigator, or designated representative, should complete the CRF pages as soon as possible after information is collected, preferably on the same day that a trial subject is seen for an examination, treatment, or any other trial procedure. Any outstanding entries must be completed immediately after the final examination. An explanation should be given for all missing data.

The completed CRF must be reviewed and signed by the investigator named in the trial protocol or by an authorized sub-investigator. To ensure that the database reproduces the CRF correctly, the Institute of Medical Biometry and Informatics Heidelberg (IMBI) accomplishes a double entry of data. Completeness, validity and plausibility of data are examined by validating programs, which thereby generate queries. The investigator or the designated representatives are obliged to clarify or explain the queries. At the end of the trial, the principal investigator will retain the originals of all CRF.

The data will be managed and analyzed in the joint unit of SDGC and IMBI in accordance with the appropriate standard operating procedures (SOP).

#### Trial monitoring

Monitoring is carried out in accordance with ICH E6 (GCP) and standard operating procedures of the Coordinating Centre for Clinical Studies (KKS) Heidelberg. Two different monitoring strategies are used within as the HASTA trial is part of the ADAMON project (Prospective cluster-randomized study of trial-specific adapted strategies for on-site monitoring in combination with additional quality management measures [11]), which is funded by the German Federal Ministry of Education and Research (BMBF, 01 EZ 0876).

#### Risk-adapted monitoring strategy

Half of the trial centers, chosen at random, are monitored by a risk-adapted monitoring strategy which is described in a trial specific monitoring manual. Participating centers are activated with an initiation visit by the monitor, who will hand-out the prepared investigator site file. All relevant trial issues are discussed and personnel are trained on trial specific procedures, documentation and web-based randomization. The monitor is in regular contact by phone or e-mail with all participating centers to follow progression of the study, protocol adherence, and to discuss trial related problems. Further monitoring visits are carried out during the course of the trial for source data verification of relevant core data, i.e., patient informed consent, inclusion/exclusion criteria, performed treatment, primary and secondary endpoints and serious adverse events. Frequency of regular monitoring visits depends on the number of recruited patients and the performance of each trial center. Every trial center is visited at least once during the trial. Close-out visits are not as a standard foreseen in the centers.

#### Full monitoring

The remaining participating centers will be monitored by a "full" or 100% monitoring (control-intervention for ADAMON) including: initiation visit, first regular monitoring visit (after inclusion of first patient), further regular monitoring visits (after inclusion of 8 additional patients or every 6 months, if new patients have been recruited), 100% source data verification for all included trial patients, close-out visit.

In addition, an efficient central supervision of the clinical trial is established (central monitoring) for both strategies. Investigators in the participating centers will support the monitor in his/her activities.

#### Audits

A final audit is scheduled in the participating centers. These audits are carried out to check whether the conduct of the study is in accordance with ICH-GCP regulations. Independence from the trial personnel involved in HASTA is guaranteed for every auditor. Investigators have agreed to give auditors free access to all relevant documents. Based on the results of these audits the two monitoring strategies will be compared.

#### Assessment of safety

According to ICH-GCP the term "adverse event" covers any clinically relevant sign, symptom, syndrome, illness that appears or worsens in a subject during the period of observation in the clinical trial and that may impair the well-being of the subject.

Adverse events fall into the categories non-serious and serious. Non-serious adverse events will not be documented in HASTA trial. The following conditions and treatments are expected after the initial operation and will therefore not be classified as AE: pain, nausea, hyper-/hypotension, blood sugar problems, electrolyte imbalances and other lab values out of normal range, if they are not exceeding the duration and extent that can be expected after such an operation.

However, all endpoint relevant complications (i.e. bowel obstruction, wound infection, anastomotic leakage of the ileostomy closure, re-operation) are explicitly being asked for and documented in the CRF as endpoint (not as adverse events). Any other complications that are considered as clinically relevant by the investigator should be documented in free text.

From the day the subject has signed informed consent until the regular end of trial at 12 months follow-up or until premature withdrawal of the patient, all serious adverse events (SAE) must be documented on a "serious adverse event form" available in the investigator site file. Serious adverse events have to be reported by the attending physician to the principal investigator within 5 days after the SAE becomes known.

It is the responsibility of the principal investigator to register all SAEs and to check incoming SAEs as to completeness, correctness and plausibility.

In case of any irregularities for example concerning the frequency or type of SAE reported the principal investigator will inform the members of the Independent Data Safety Monitoring Board (DSMB) without delay. At least once every 12 months, the DSMB will receive a written safety report. The members of the DSMB then report the result of the benefit/risk assessment to the principal investigator and will give appropriate recommendations concerning the continuation of the trial.

Analysis of safety related data is performed with respect to frequency of SAE in both treatment groups and frequency of SAE stratified by causality.

### Statistical methods

#### Analysis

##### Analysis sets

Each patient's allocation to the different analysis populations (full analysis set (FAS) according to the intention-to-treat (ITT) principle, per protocol (PP) analysis set, safety analysis set) will be defined prior to the analysis. The allocation will be documented in the statistical analysis plan. During the data review, deviations from the protocol will be assessed as „minor" or „major". Major deviations from the protocol will lead to the exclusion of a patient from the PP analysis set.

##### Confirmatory analysis

The null-hypothesis is assessed by testing the intervention effect in a logistic regression model that takes into account the covariates "intervention" (hand-suture/stapler) and "skill of surgeon" (board certificate yes/no). Due to the high number of targeted centers (approximately 20), the variable "center" for which randomization was stratified is not planned to be additionally included in the model. A two-sided type I error rate of will be applied.

Confirmatory analysis will be primarily based on the FAS which is consistent with the intention-to-treat (ITT) principle by including all patients who were randomized to the two groups. This approach reflects the idea that the study should match as close as possible to the conditions in clinical practice.

If a patient discontinues from the study prematurely, missing data with respect to the primary outcome variable will be replaced by ICA-r method described by Higgins et al. (2008) [12].

##### Further analyses

In addition to the evaluation of the FAS, a PP analysis will be performed including all randomized patients without major protocol violations.

The secondary variables will be analyzed descriptively by tabulation of appropriate measures of the empirical distributions, descriptive *p*-values for treatment group comparisons and associated 95% confidence intervals. Possible center effects will be analyzed, too. All additional evaluations will be described in the statistical analysis plan, which will be fixed prior to database closure.

#### Homogeneity of the treatment groups

The homogeneity of the treatment groups will be described by comparison of the demographic data and the baseline values.

Data management and analysis will be performed using SAS, version 9.1 or higher.

#### Criteria for termination of the trial

The principal investigator has the right to terminate the trial and to remove all trial material from the trial center at any time in consultation with the trial statistician and the steering committee. For any questions concerning safety of trial subjects the DSMB should be consulted.

Reasons that may require trial termination include potential health hazard caused by the study intervention and indicated by the prevalence or severity of adverse events, unsatisfactory patient enrollment with respect to quality or quantity or data recording is severely inaccurate or incomplete. Also, new external evidence may necessitate termination of the trial.

## Trial organization and administration

There are several institutions that ensure safety, transparency and reproducibility of the trial. The steering committee consists of eight independent members (surgeons, clinical investigators, biostatisticians). Tasks of the steering committee are review of the trial protocol before the beginning of the trial and evaluation of Data Safety Monitoring Board (DSMB) recommendations regarding premature study discontinuation. The DSMB consists of three independent members (surgeons, biostatistician). In case of any irregularities for example concerning the frequency or type of SAE reported the principal investigator will inform the members of the independent DSMB without delay. At least once every 12 months, the DSMB will receive a written safety report. The members of the DSMB then report the result of the benefit/risk assessment to the principal investigator and will give appropriate recommendations concerning the continuation of the trial.

### Investigator meeting and training

78% of centers participating in the HASTA trial are non-university hospitals coming from the organization of certified German bowel centers, some of them with little trial experience. Therefore, we organized a 2-day investigators meeting with following topics on the agenda: introduction to the trial protocol and its rationale, discussion of surgical procedures, preparation for study initiation and patient documentation. All partners from the trial management presented their responsibilities: biometry, data management, monitoring, project management and surgical coordination. To stan dardize surgical procedures, an operative training session was held in a special surgical laboratory. Participants were thus given the opportunity to practice surgical techniques for stapling and hand-suture. 17 out of 18 participants returned the SDGC questionnaire for evaluation of content and rhetoric of the speech as well as for the printed handout, giving a rating between 1.2 and 2.2 (1 is the best end and 6 is the worst end) to all speakers. Yet, the operative session was unanimously considered the highlight of the meeting. Participants also commented very positively that ample time was given for discussion and in question and answer sessions. Thus the meeting was very well accepted by all participants (see general evaluation table 2) and was successful in forming a cooperative trial group.

**Table 2:** Evaluation of the investigators' meeting

	1 = exactly applies (N)	2 = rather applies (N)	2 = does rather not apply (N)	3 = does not apply at all (N)	Median
Communication of content was precise and comprehensive	**10**	**5**			**1.3**

The meeting was well-arranged	**14**	**2**			**1.1**

Content was communicated on the meeting as announced	**12**	**3**			**1.1**

There was a relation between theory and practice	**10**	**6**			**1.3**

The use of media was helpful for understanding	**9**	**7**			**1.4**

The schedule and duration was adequate	**8**	**6**	**2**		**1.6**

The learning success was supported by repetition and summary	**4**	**12**			**1.7**

Questions were answered in an understandable way	**12**	**4**			**1.3**

The meeting had a thread	**10**	**6**			**1.4**

The meeting augmented my state of knowledge	**6**	**5**	**5**		**1.9**

The meeting met my expectations	**8**	**8**			**1.5**

I agree with the study rationale	**8**	**8**			**1.5**

The study rationale is justified	**9**	**6**	**1**		**1.5**

The primary endpoint is plausible	**8**	**8**			**1.5**

I am able to perform the surgical procedures according to the protocol	**10**	**2**	**1**	**1**	**1.5**

I was comfortable with the accommodation and social program	**10**	**6**			**1.4**

### Ethics

This study is accomplished according to the Helsinki Declaration in its latest version dated 2004, the Medical Association's professional code of conduct and the international principles of the Good Clinical Practice (ICH-GCP). The trial will also be carried out in compliance with national legal and regulatory requirements. The medical secrecy and the German Federal Data Protection Act will be observed. After termination the trial will be reported in accordance with the CONSORT statement [13].

Patients receive complete oral and written information on the trial by a medical doctor and a written informed consent has to be signed.

Before the start of the trial, the clinical trial protocol, informed consent document, and any other appropriate documents had been submitted to the independent ethics committee (IEC). The documents were approved by the independent ethics committee of the University of Heidelberg Medical School on September 23, 2008. Secondary approval is sought from all local ethics committees of the participating centers. The IEC must be informed of all subsequent protocol amendments. Amendments must be evaluated to determine whether formal approval must be sought and whether the informed consent document should also be revised. The investigator must keep a record of all communications with the IEC. The trial was registered by the German Clinical Trial Register (http://www.germanctr.de/index_de.html) with a unique identification number (DRKS00000040) on October 27, 2008.

The trial management is committed to writing a scientific publication in any case, even if the trial is stopped early. The design of the trial and the trial results will be published and the authorship will be assigned by the trial management. Representatives of the four highest recruiting trial centers will act as co-authors and each participating center will be mentioned.

**Figure 3:**
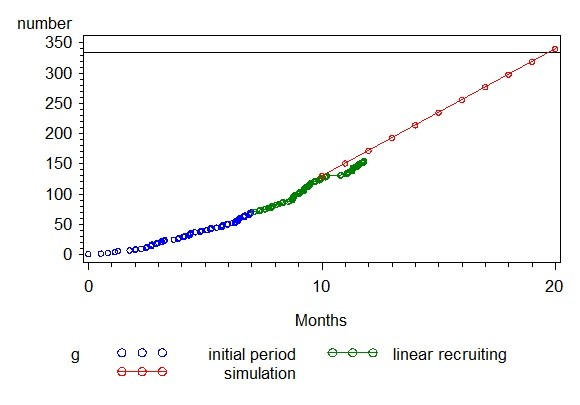
**HASTA patient recruitment**.

The first center was initiated in January 2009, the first patient was randomized in February 2009. 334 patients should be randomized within 20 months. After a follow-up period of 12 months the trial should be terminated in October 2011. Figure 3 shows the planned versus the actual recruiting rate within the HASTA trial.

## Discussion

If there are two or more treatment options for one clinical condition a randomized, controlled trial with a clinically relevant endpoint should determine which is more beneficial to the patient [14]. Concerning closure of loop ileostomy after low anterior resection we are currently observing a situation of clinical equipoise as there is only one small, mono-center trial postulating that stapled anastomosis is superior compared to hand-sewn anastomosis regarding bowel obstruction. So we decided to design a multicenter randomized, controlled trial with a sufficiently powered sample size to answer this question. Volume of documentation is restricted to essential and relevant variables and should result in high acceptance and little loss of data. In special conditions requiring very complex treatments it may be preferable to standardize the procedure and to choose specialized centers as participants of the trial meaning an experimental design. As closure of loop ileostomy is a common procedure performed by community hospitals as well as by referral centers we chose an individual design in terms of a pragmatic trial. The trial should reflect the reality in the treatment of this condition. This is why the technique of performing the hand-sewn anastomosis may be done according to local standards. Learning a certain technique of hand anastomosis results in a learning curve which impairs the results of the trial [10] and we did not want to display learning curves in this trial. The participants are free to perform their routine treatment (i.e. use of drainage, abdominal wall closure etc.) but their practice needs to be documented and will be reported. In contrast a stapled anastomosis can be standardized easily across all centres. To minimize performance bias the exact technique was taught to all participants at the investigators meeting and opportunity was given to practice in a wet lab. To avoid selection bias, we have reduced inclusion and exclusion criteria to a strict minimum. Participating centers have to maintain a screening log documenting all potential patients. Due to the nature of a pragmatic trial, special attention is given to primary and secondary endpoints that are essential for the assessment of both techniques. The primary endpoint bowel obstruction is relevant from the patient perspective as well as from a health care perspective. Bowel obstruction often requires prolonged hospitalization or re-hospitalization of the patient. No general definition of the term "bowel obstruction" is available neither does a consensus statement exist. Therefore, it was defined according to the prior trial to maintain comparability and allow further pooling in a meta-analysis. In addition, the given definition can be monitored easily from the source document, i.e. the patient's chart and thus observer bias can be reduced.

## Abbreviations

AE: Adverse Event; BMBF: German Federal Ministry of Education and Research; CRF: Case Report Form; DGCH: German Surgical Society; DSMB: Data Safety Monitoring Board; FAS: Full Analysis Set; GCP: Good Clinical Practice; ICH: International Conference on Harmonisation of Technical Requirements for Registration of Pharmaceuticals for Human Use; IEC: Independent Ethics Committee; IMBI: Institute of Medical Biometry and Informatics; ITT: Intention-to-treat; KKS-HD: Coordinating Centre for Clinical Trials - University of Heidelberg; LAR: Low anterior resection; PP: Per Protocol; RCT: Randomized Controlled Trial; SAE: Serious Adverse Event; SDGC: Study Center German Surgical Society; USP: United States Pharmacopoea.

## Competing interests

All authors disclose any financial and personal relationships with other people or organizations that could inappropriately influence their work within this project. The authors declare that they have no competing interests.

## Authors' contributions

TL and CMS participated in the design of the study, wrote the manuscript and had full access and responsibility for all data. IR coordinated the trial and participated in writing the manuscript. TK, OT, RH, MK and TS contributed to patient accrual and helped to draft the manuscript. TB and MK participated in the design of the study, perform the statistical analysis and helped to draft the manuscript. MB supervised the study conduct and revised the manuscript draft. JW conceived the study idea and participated in writing the manuscript. All authors read and approved the final manuscript.
